# Typho-Malarial Fever

**Published:** 1892-06

**Authors:** J. W. Duncan

**Affiliations:** Atlanta, Ga.


### *ORIGINAL COMMUNICATIONS.*

### TYPHO-MALARIAL FEVER.*

BY

J. W. DUNCAN, M. D.,

ATLANTA, GA.

Typho-malarial fever is a disease about which very little has been written so far as I have observed. Its name implies that it is a compound disease, therefore its causes must be multiple. The etiology may be considered under the heads of, first, predisposing, and second, exciting causes. There is perhaps no age at which a person can be said to be exempt from the disease, but the predisposition is greater in childhood and early adult life than in middle life and old age.

Seasons have a marked influence in producing an increase or diminution of the fever. It is much more common in summer

*Read before the Georgia Medical Association, April 22, 1892.

and autumn, than in winter and spring. Hot weather in connection with a dry season will, perhaps, be attended with an increase of the malady above that of a dry season and low temperature ; but the dry weather seems to have more to do in the production of the disease than the heat. I believe that the poison or agent which produces the typhoid element in the disease is rendered potent by dry weather, whether it be a living germ or not and that it is rendered innoxious by frequent rains. My observations have been that the disease is more common and severe in those sections where the surface of the earth dries quickly than where the soil is less porous.

As regards the malarial element in the disease, I know there have been a number of theories as to its etiology, yet, so far as I am informed, no chemist has been able to demonstrate the existence of malaria. Yet that which is worth far more to the practitioner than any theory, the fact of its existence from its effect upon the human organism, stand up before us and we cannot deny its entity, although it has baffled the skill of the chemists with all his analytical resources. It is generated in greatest abundance in marshes, which contain a high percentage of organic matter, hence the name marsh miasm. Notwithstanding the above name it is often observed to manifest itself in quite a variety of of soils and climates, and has even been quarried out from its pent up bed in the granite rocks of Hong Kong. From some observations it does not seem necessary that there be surface moisture to produce the poison, but sub-moisture, even under a very dry surface especially, if the soil be disturbed or thrown up during warm weather, or if a forest be removed so as to allow the sun to dry the surface, while the substratum is still moist, the noxious agent, with such conditions, will manifest itself upon the human organism. Decaying matter, heat and moisture are believed to be in a sense essential in the production of the poison.

We are forced to acknowledge that the elements essential to the production of the toxic agent are found in many parts of the world. I do not claim that the topography, climate and soil of this State is a garden prolific in the generation of the miasmatic germ, but I am fully convinced that there is a sufficient amount of miasmatic poison generated here to produce a toxic influence upon the human system, and whether it manifests itself in intermittent or remittent fever or not, whatever the ailment may be, while the system is thus impressed with miasm, periodicity is sure to show itself.

I do not believe that the agent which is capable of producing such curious impress of periodicity, as is observed in malarial fevers and the septic agent, or bacillus, which produces typhoid fever, are ever united outside the human system into a compound third agent, capable of producing typho-malarial fever; but the malarial element may exist in the system before the typhoid agent enters, or they may enter simultaneously.

I do not propose to enter into an argument on the nomenclature in this paper. The disease has had quite a variety of synonymns, for instance, "pseudo typhoid," "gastric fever," "nervous remittent," "continued remittent." These and many other names have been applied to the peculiar compound disease which I am trying to describe. Typho-malarial fever is the name which I believe to be the most appropriate, and it has been adopted by quite a number of the profession since the late war between the States.

The camp fever of the army of the Potomac was generally recognized as a typho-malarial fever, in which symptoms of typhoid fever, diarrhœa, rose rash, etc., were associated with those of malarial fever. Contagion is supposed to be among its exciting causes by some of the profession and many of the laity, but I do not believe that it is contagious in the sense that measles, whooping cough, smallpox, etc., are ; but those who are almost constantly in the sick chamber, and who are perhaps necessarily exposed to the same zymotic influences, which caused the disease in the first case or cases, are very likely to succumb to these influences. Yet it must be admitted that the occurrence of any number of cases simultaneously, or successively, in any house or neighborhood can never prove the fact of contagion.

I am inclined to believe in the spontaneous origin of the disease wherever sanitation is wholly or in part neglected, and notwithstanding the sanitary regulations of our cities and the noble efforts of our city officials to enforce the same, we may find dung hills, animal and human, accumulated or pent up sewage, cess-pools, contaminated wells, decaying vegetables, poorly constructed basements, and last, but not least, in the *heat* of *summer* streets torn up from one to ten feet in depth, along many of our populous thoroughfares. These things cause an effluvium to arise and spread over the people which, if it cannot be felt, can be smelt.

It may be that the electric state of the atmosphere is so modified by these causes that we are called upon to give up a part of our nervous force, and thus our equilibrium is destroyed; but I am inclined to believe that the poisons enter the system by inhalation, absorption, and ingestion, and having gained admission, the enemy attacks the nervous centers per se, especially the sympathetic, and its depressing influence on the great ganglionic system produces the secondary local manifestations, as observed in the enlarged, softened, and later on ulcerated solitary glands, enlarged liver and spleen, and these, together with the poisoned nervous centers, bring about the train of constitutional symptoms. Dr. C. Coleman Benson, of Baltimore, Md., says from observation based upon hypothesis: "I feel assured that the irritation and pathology of all fevers lie in the excessive activity of the large ganglionic cells of the gray matter of the anterior cornua of the spinal cord, and of the cardiac and respiratory centers, in the medulla oblongata, due to the disturbed electric balance of the sympathetic and spinal nervous system, and that fever or rise of temperature is but a symptom of that nervous condition."

In support of this view he says: "Irrespective of my recent clinical success, temperature is controlled by motor depressants, as aconite, veratrum, quinine, etc., but the effects of the fever are the serious points for treatment, and those which generally baffle the practitioner and lead to a fatal issue."

When the temperature does not exceed 101° F., the disturbance is chiefly in the sympathetic nervous system, and indicates the first stage of irritation in it, and in the cord and centers of the medulla; but when the temperature exceeds 102.5° F., then the sympathetic system has become paralyzed, and the arterial circulation is left to the control of the ganglionic centers of the cord and medulla, by which these centers become engorged with blood and paralyzed in their functional action on the muscular and other tissues and glands of the body.

In this condition the tissues are devitalized, undergo chemical changes of combustion by oxidation, which leads to their waste, and when the wasting has reached the point of starvation, then the brain becomes involved, and delirium is established.

The diagnosis requires that there shall be present in the case, in a degree, the local and constitutional manifestations of typhoid fever, associated with malaria or periodicity, one of its leading phenomena.

The attack is more often abrupt, and more frequently attended with a chill, than in simple typhoid. There is greater tendency to biliousness. The temperature in the first week rises higher than in the non-malarial.

There is more gastric disturbance and less tendency to delirium than in simple typhoid. There is also a greater tendency to the accumulation of effete material. There is usually less diarrhœa and tympanites than in the unmixed disease. Relapses are more frequent and less severe.

There are greater variations in the daily temperature than in typhoid fever. The tongue is more serrated at the edges, and less disposed to be red, and more to be coated, than in the pure typhoid type.

During the first five or ten days there are periodic remissions and exacerbations, accompanied with coolness of the extremities, and if nothing be used to interrupt these remissions and exacerbations they may continue throughout the entire course of the disease. The duration of the disease is from two to six weeks.

There may be epistaxis during the first or second week, but the hemorrhage most to be dreaded is from the intestines, which takes place, if at all, late in the disease. I have observed it even after convalescence seemed to be very well established. The prognosis, in consequence of such accidents or complications, must be formed or expressed with great caution. The mortality, however, is not as great as in typhoid fever. Whether this be due to an antagonistic influence of the two toxic elements on each other, or to a more rational therapeutics, it is difficult for me to decide ; at any rate, the disease is more amenable to treatment.

Pneumonia occasionally develops in the course of the disease, especially the prostatic variety ; and owing to the nervous system being obstructed, the pneumonia may make considerable progress before it is discovered, unless we institute physical examination from slight premonitions of the disease. Inflammation and even suppuration of the parotid glands have been observed in a number of cases, and when the parotid glands are involved there is some danger of a metastasis to the testicles.

When the parotid glands do suppurate, the pus is unhealthy, thin and ichorous, and is disposed to disseminate itself in the gland and adjacent cellular tissue, and in some cases it is absorbed and carried into the general system, greatly increasing the septic condition.

*Treatment.*—In the early stage of the disease, while there are headache, backache and aches generally, I like the effect of salicylate of soda, salol or phenacetine. Quinine acts well in equalizing the circulation, and aids in preventing the cold stage, and for this purpose the sulphate or bi-sulphate may be given in five or ten grain doses, once or twice in the twenty-four hours ; but I am not in favor of using it in large doses with a view to its antipyretic effect, for I have seen patients deranged for hours by large doses given for this purpose.

Tinct. veratrum viride, tinct. aconite, fl. ext. gelsemium, either one alone or all combined, act well in lowering the temperature in the early stages.

The coal tar preparations, antifebrin, antipyrin, phenacetine, etc., in small doses repeated *pro re nata*, have a very decided effect in lowering the temperature, but great caution must be used in their administration. I consider phenacetine the least depressing of any that I have used.

Frequent sponging with tepid water is useful and very grateful to the patient. I have used the wet pack in a number of cases with good results. Where there is dryness of the mouth and great thirst I have used fl. ext. jaborandi with good results.

To revive the action of the liver and improve the secretions generally, mercurials may be used more freely and frequently than in simple typhoid fever; and I believe that the bichloride of mercury, from its antiseptic properties, may be given with a view of accomplishing more than a simple action on the secernent system.

I have given turpentine emulsion very rarely for a number of years. I use turpentine locally over the abdomen in the form of stupes, etc., and believe that it is very useful. I have used the comp. tinct. iodine, tinct. mur. iron, pure pepsin, cinnamon water and glycerine. The pepsin aids digestion, while the iron and iodine form a valuable antiseptic tonic and stimulant. I was led to use the above combination in typho-malarial fever, from having treated successfully chronic intermittent fever with it. Opiates are often indicated to procure rest and check diarrhœa.

Flushing the bowels once daily with tepid water, from one to two quarts in which ten grains of salicylate of soda and sixty grains of boric acid have been dissolved, helps to prevent diarrhœa and tympanites. Where headache is severe bromo-caffein acts well and has a soothing effect on the stomach and nervous system.

Tinct. digitalis improves the action of the kidneys and gives tone to the heart. When the pulse is frequent and feeble it may be given to increase the force and lessen the frequency. When tympanites is present sulphite of soda and charcoal, either alone or combined, may be given with great benefit, and also enemas containing tinct. assafœtida are beneficial.

When alcoholics are used the purest wines and brandies should be selected. The effect of the stimulant and the condition of the patient should govern us as to the quantity to be given in the twenty-four hours.

Hemorrhages and other complications should be managed just as they are in typhoid fever.

The diet should be principally fluid, consisting of milk, soup and meat essences. The patient should be encouraged to take as much nourishment as he can appropriate without inconvenience, as it will sustain his strength and help to prevent the ultimate fatal collapse, which should be guarded against in all asthenic diseases. We should insist upon his having nourishment systematically if not scientifically. If the patient be rational and express a constant desire for any article of diet, his craving must be regarded as a demand of nature, in which case he may be permitted to eat that which is ordinarily interdicted, and will do so, if allowed, with apparent benefit.

During convalescence I have in some cases given the comp. syr. hypophosphites, and I believe that it has been beneficial. I quote again from C. Coleman Benson. He says: "My specific for all temperatures from 101° to 107° F. and dependent symptoms is phosphorus, which should be given in temperatures from 99° to 101° in doses of $\frac{1}{150}$ of a grain every half hour for six doses, and then every two hours during the day, till reduction of temperature to normal. If the temperature vary from 101.5° to 107°, then give $\frac{1}{100}$ of a grain every half hour for four doses, and then every two hours during the day till reduction in temperature to normal, never, however, exceeding in the twenty-four hours $\frac{1}{3}$ grain, and even less if epigastric pain be produced by it. The temperature at first will rise slightly, but descend rapidly from 3° to 4°. When the normal has been attained and remained so for twenty-four hours, then give $\frac{1}{100}$ of a grain, with food thrice only in the day."

While I admit that phosphorus is a very excellent nerve tonic and stimulant, acting on the circulation through the nervous system, causing the pulse to be fuller and more frequent, distending the capillaries until free perspiration follows, and from its primary action increasing the heat of the surface of the body slightly, its secondary effect lowers temperature. It accelerates cell growth in the body. It acts as a diuretic. The urates and urea are greatly increased in the urine. Yet I do not think it should be considered a specific for fevers. It may act well as an auxiliary in the management of some cases, and doubtless it does. But I think that antiseptics should form the basis of treatment in all cases which have sepsis as the exciting cause. While it may not play an important part in causing the fever under consideration, it does serve as the *materies morbi* in many cases of sickness attended with high temperature.

We should not allow ourselves to be led astray by hobbies and specifics, so-called, but should try to meet the indications in each individual case. It is well known that pyrexia results from various causes ; therefore, we should select our remedies with the view of removing the cause as well as relieving the symptoms.

The disease under consideration, as well as many others, is classed as self-limited ; therefore it is claimed that nothing can be done to shorten the duration. We can only use placeboes and look wise, and wait for the *vis medicatrix* nature to do the rest. But I believe the time will come when many of the so-called self-limited diseases will be greatly abridged, if not aborted. It is well known from clinical experience that pneumonia has often been cut short in the first stage, and I believe that remedies can be used in typho-malarial fever that will very much aid in the elimination of the poisons from the system, if not in some degree neutralize them.

Our object in every case and class of disease should be to aid nature. Every person who is exposed to the bacilli of typhoid and the miasm of malaria does not succumb to them ; but nature, ever true to herself, neutralizes, eliminates and subjugates them, and man, the grandest being on earth, moves on notwithstanding the pestilence.

